# Influences of Augmented Reality Assistance on Performance and Cognitive Loads in Different Stages of Assembly Task

**DOI:** 10.3389/fpsyg.2019.01703

**Published:** 2019-07-24

**Authors:** Zhen Yang, Jinlei Shi, Wenjun Jiang, Yuexin Sui, Yimin Wu, Shu Ma, Chunyan Kang, Hongting Li

**Affiliations:** Department of Psychology, Zhejiang Sci-Tech University, Hangzhou, China

**Keywords:** AR, assembly task, commissioning subtask, joining subtask, performance of the assembly task, cognitive load

## Abstract

According to the assembly task model proposed by Stork and Schubö (2010), the assembly task is divided into commissioning and joining subtasks. Each subtask includes two sequential stages, namely, perception and response selection, and action. This division enables a convenient discussion of the influences of Augmented reality (AR) assistance on operators during different stages of an assembly task. Research results can provide a basis for the further analysis of the influence mechanism of AR assistance on an assembly task. This study is composed of three experiments. Experiment 1 explores the influences of AR assistance on the performance of the overall assembly task and the commissioning and joining subtasks. Combining a variation of task complexities, Experiments 2 and 3 discuss the influences of AR assistance on the different stages of the commissioning and joining subtasks. We found that AR assistance can shorten the time of the overall assembly task and subtasks (commissioning and joining) and reduce mistakes during these tasks. Moreover, AR assistance can decrease cognitive load in the commissioning subtask, but it increases cognitive load in the joining task with low complexity. In the perception and response selection stage of the commissioning and joining subtasks, AR assistance can shorten the time for users to recognize the target part and understand the assembly relation. This advantage is extremely significant for the high-complexity task. In the action stage of two subtasks, AR assistance can shorten the time for users to capture parts, but it prolongs the time for users to build parts.

## Introduction

In the manufacturing industry, numerous assembly tasks must be accomplished manually, especially for highly customized or highly complicated products. However, humans have limited information processing capability, and the performance of manufacturing systems continuously increase, thereby increasing work complexity. Positively supporting users in using appropriate assistance systems can reduce the complexity of tasks, maintain smooth work processes, and prevent errors (Stork and Schubö, 2010; Gorecky et al., 2011). Therefore, designing an interface that will assist a manual assembly operator is important for supporting workers in the manufacturing industry (Zaeh et al., 2009; Leu et al., 2013).

Augmented reality (AR) is a system that integrates virtual objects produced by computers with the actual environment. AR superposes information produced by a computer into the real-world environment (Cheng and Tsai, 2012), and it has the characteristics of virtual reality combination, real-time interaction, and 3D registration (Azuma, 1997). In addition, information display and image superposition are characterized by situational perception, thereby implying that the presentation of virtual information is based on the object observed by users (Azuma, 1997; Azuma et al., 2001). This new technology can cooperate with human ability to provide effective and complementary tools that assist in manufacturing tasks (Lamberti et al., 2014). Therefore, AR is one of the most beneficial applications in the traditional manufacturing and assembly field (Nee et al., 2012).

### Research on AR Assistance in the Assembly Field

Although researchers differently define the composition of assembly tasks (Neumann and Majoros, 1998; Johnson and Proctor, 2004; Stoessel et al., 2008; Zaeh et al., 2009), most of them agree that an assembly task is a complicated task composed of information activities related to cognition and working activities related in manual operation. Therefore, some researchers state that to improve the performance of assembly tasks, the people’s way of thinking and use of guidance information must be changed, and information and working activities must be combined, thereby increasing efficiency of information access (Hou et al., 2015). AR technology inserts digital information into actual working spaces, thereby helping operators accurately accomplish assembly procedures and increase working precision (Wang and Dunston, 2006a, b). Integrating information and working activities can also help operators reduce head and eye movements (Tang et al., 2003) and release hand occupation (Wang and Dunston, 2006a). For vivid and adaptive display of information, AR technology can overcome the restrictions of traditional computer-assisted assembly systems compared with paper manuals. AR technology enhances operator–system interaction by allowing operators to move and operate objects without shifting attention between instructions and actual objects in an AR environment. This approach has been viewed as the solution to interaction problems for virtual and real objects (Azuma et al., 2001).

The advantages of AR in the performance and mental load of assembly tasks compared with other assisting media have been proven by studies. Researchers have simulated assembly tasks by using building blocks and 3D puzzles in the experiments to study the influences of AR assistance on assembly tasks. Tang et al. (2003) evaluated the validity in the assembly of Duplo building blocks with the assistance of AR. In this study, the assembly error under the use of head-mounted-display (HMD) AR decreased by 82% compared with the assembly error obtained with guidance information displayed on a common computer screen or printed on paper. Hou et al. (2013, 2015) conducted an empirical study on animations in the AR assistance system of the Lego scientific model and for pipeline assembly. Results of both experiments showed that animations in an AR assistance system can shorten the time for accomplishing tasks and reduce assembly errors and overall cognitive loads compared with paper manuals. Moreover, certain operators positively evaluated AR assistance systems. Siltanen et al. (2007) proposed a multimode AR interface for assisting manual assembly. This system provides voice and gesture interfaces for interaction with operators. In the research, the users accomplished a simplified puzzle task with 3D building blocks, and the qualitative data collected by the author reflected the advantages of the AR assistance system.

Some studies have reported the role of AR assistance in the maintenance and general assembly of traffic tools and medical equipment (Neumann and Majoros, 1998; Wiedenmaier et al., 2003; Nilsson and Johansson, 2007), assembly of parts in complex industries (Boud et al., 1999; Henderson and Feiner, 2011; Wang et al., 2016b), and assembly tasks of medium- and small-sized electronic products (Baird and Barfield, 1999; Seok and Kim, 2008). In comparison with a group that used paper and digital document assistance on a liquid crystal display (LCD) screen, a group using AR assistance showed significant advantages in terms of task completion time and accuracy. In assembly training, the effect of AR technology assistance has been verified by studies (Gavish et al., 2011; Webel et al., 2011a, b). Operators, who are trained by the AR assistance system, perform efficiently in an actual assembly task and hardly make mistakes (Sabine et al., 2013).

Most of the abovementioned studies demonstrate the influence of AR assistance systems by evaluating the overall performance of assembly tasks. Although most studies demonstrate that AR technology is beneficial for assembly performance, no clear and reliable conclusion has been drawn about the specific assembly procedure for which this technology works.

### Information Processing Framework of Assembly Tasks

In an AR assistance system, understanding the cognitive process of the assembly procedure is a prerequisite for appropriately using the situational data provided by the AR technology (Stork and Schubö, 2010).

Stoessel et al. (2008) studied the cognitive process and possible cognitive bottlenecks in an assembly task. They believed that the focus should be on three aspects: selective visual attention, multitask performance, and mental load. To address these problems, the authors appropriately organized the cognitive processes involved in manual assembly by using an information processing framework. This framework is based on the concept that environmental stimuli are accepted by human organs and can be processed continuously through several determined stages until one responsive action to the stimuli is finally produced. In general, the details of guidance information must be detected and recognized in an assembly task and then transformed into appropriate action responses, which must be implemented accurately. Therefore, this information processing framework is highly appropriate for constructing cognitive processes involved in an assembly task. That is, such framework can decompose the cognitive processes involved in manual assembly into several substeps. On the basis of these studies, Zaeh et al. (2009) and Stork and Schubö (2010) further refined the information processing framework of an assembly task. They believed that the information processing of an assembly task involves all cognitive functions, from perception, attention, and memory to action planning and implementation. First, the assembly task can be divided into two subtasks: commissioning and joining. In the commissioning subtask, participants should identify a relevant assembly part on the assembly manual and search and grasp it in a specific storage box. In the joining subtask, participants should locate instructions on the manual, process the part position and orientation, and then assemble the parts in accordance with the instruction. In summary, several assembly parts should be identified, selected, and grasped during the commissioning subtask. In the joining phase, the previously selected parts should be assembled in accordance with the instruction (Zaeh et al., 2009). Second, each subtask comprises the following stages: perception and response selection and action. These stages occur in order.

**FIGURE 1:**
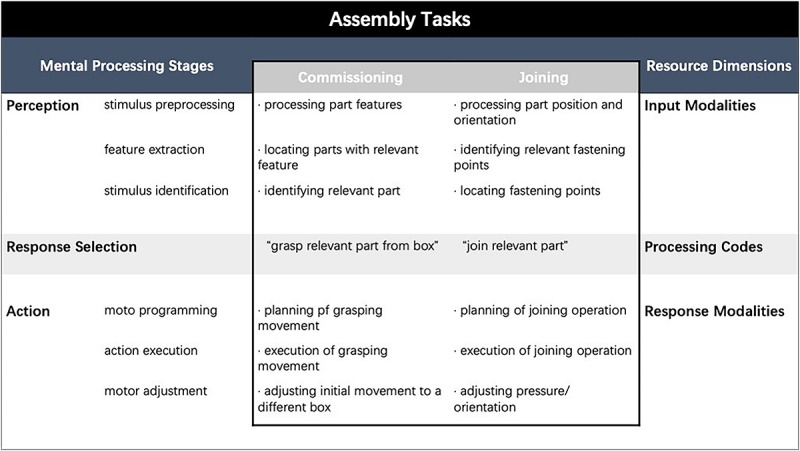
Information processing framework of assembly tasks.

Figure 1 shows the information processing and essential resource dimensions in the two subtasks (Stork and Schubö, 2010). The processing stages involved in the task are implemented in order. Multiple resource information processing theory indicates that some subtasks might interfere mutually due to the limited capacity of psychological resources. Task performance declines accordingly when two subtasks require the same resources (e.g., when parts are captured by the left hand and assembled by the right hand or when visual shifts are performed in the manual, part, and assembly zones).

The information processing task is conducive to understanding the source of artificial errors committed during assembly. Errors are caused by inadequate resources or inappropriate distribution due to limited psychological resources, which should be scattered or distributed to different psychological processes.

On the basis of such division, Stork et al. (2008) performed a series of studies to explore the influences of AR assistance on assembly tasks. One study calculated the total time required to complete two subtasks (total time for commissioning and joining) and analyzed the movement parameters of users (Stork et al., 2008). In the experiment, the task assembly performance with the assistance of projection display, LCD, and contact analog highlighting display was compared. The findings showed that the participants under the assistance of contact analog highlighting display, which is an AR display, started to capture objects significantly earlier and completed the action more rapidly than those under the assistance of LCD. Moreover, the peak speed and peak acceleration of the capture action are remarkably higher than those under the assistance of LCD. Thus, the participants assisted by AR display accomplished the action stage of the commissioning subtask rapidly. In another study, Stork et al. (2009) measured the completion time of the commissioning subtask and eye movement parameters. Experimental results demonstrated that under exogenous clue (highlighted project position), the results of competition time and gazing number in the commissioning subtask were significantly superior to those obtained with the assistance of projection display and LCD. The series of studies by Stork et al. (2008, 2009) focused on the commissioning subtask and did not involve the joining subtask in the experimental task design and data acquisition. Moreover, these studies did not adjust the complexity of the commissioning subtask.

Wang et al. (2016b) believed that this assembly task model is convenient for decomposing the cognitive process of a task into different stages. They proposed an interactive AR-assisted assembly system based on human cognition. Moreover, with reference to the division method of the cognitive process in the assembly task used by Stork and Schubö (2010), the experimental task was divided into three stages: understanding of guidelines, recognition of specific components, and assembly. Many assembly guidelines, such as text, 3D rendering images, attention-oriented augmentation (e.g., highlighting), and assembly animation and dynamic paths, were provided in accordance with the characteristics of each stage. The experimental results showed that such system based on user cognition achieved significantly shorter competition time and higher accuracy in recognizing specific components and implementing assembly than the traditional AR assistance system and LCD-based digital document system. In addition, the ratings of the overall user experience were improved. Hence, Wang et al. (2016b) believed that the practicability and effectiveness of the model were validated by their studies.

Although the assembly task model of Stork and Schubö (2010) was applied, the cognitive process in the model was divided in accordance with the characteristics of the applied experimental tasks, and Wang et al. (2016b) did not distinguish two subtasks in the model. That is, the model of Stork and Schubö (2010) not only divides the assembly task into two subtasks (the commissioning and joining subtask) but also further distinguishes different cognitive processes in both subtasks. Moreover, the model is combined with the Resource Theory of Wickens (2002), which divides the mental resources into four dimensions: perceptual processing, processing codes, processing stages, and response modalities. The model of Stork and Schubö (2010) proposes that the resource dimensions of the perception and response selection stage are input modalities and processing codes, and the resource dimension of the action stage indicates response modalities. Thus, this model was selected to identify the influence of the AR assistance on different subtasks and stages.

The effectiveness of AR assistance depends on the complexity of assembly tasks. Radkowski et al. (2015) presented an analysis of two factors that might influence the effectiveness of AR assistance, namely, the complexity of visual features and assembly tasks. Moreover, Wiedenmaier et al. (2003) found that AR assistance was more suitable for complex assembly tasks than the traditional paper manual, whereas for easier assembly tasks, no significant difference was found between the two support media. However, Wiedenmaier et al. (2003) used an overall assembly task and did not distinguish different subtasks and stages of the task. Hence, when the complexity of assembly task varies, whether the AR assistance differs from the paper manual in different subtasks and cognitive stages is not evident.

### Research Goal

The positive effect of AR assistance on improving assembly performance has been proven by previous studies. However, an assembly task is complicated and combines information activity related to cognition and working activity related to parts; it can be divided into different subtasks and stages. The previous studies have proven the effect of AR assistance on the overall assembly task. However, the influence of AR assistance on the performance of assembly subtasks and the different stages of each subtask remain unknown. In addition, when the complexity of the assembly task varies, the influence of AR assistance on the performance of subtasks and different stages requires further exploration. Hence, the main goal of this research is to investigate the influence of AR assistance on the different subtasks and stages of the assembly task on various complexity conditions.

The assembly task model proposed by Stork and Schubö (2010) is convenient for dividing the assembly task into different stages. The practicability and validity of the model have been proven. Therefore, this assembly task model is used as the theoretical basis of this study. Research conclusions can provide references for the refined evaluation of the effects of AR assistance on assembly tasks.

The construction of building blocks is used as a typical assembly task. In the experiment, users should place building blocks in the correct position and order in accordance with the requirements of static instruction or augmented information. In comparison with complicated assembly tasks in industrial manufacturing, this task can be adjusted in terms of quantity, order, and target shape in accordance with the experimental requirements, and the physical materials have small volume, light weight, and considerable safety; thus, they are convenient for laboratory operations. Moreover, the assembly task conforms to the hypothesis of the information processing framework; it covers two subtasks: commissioning and joining. Influences of the AR system on the assembly task can be tested, and valuable feedback on actual manufacturing tasks can be obtained through this simplified task.

Three experiments are conducted in this study. We hypothesize the following by combining the results of previous studies:

(1) In comparison with screen-based documentation assistance, AR assistance can not only significantly shorten the time for the overall assembly task and commissioning and joining subtasks but also reduce errors and cognitive loads.
(2) In comparison with screen-based documentation assistance, AR assistance can shorten the completion time of the perception and response selection stage and action stage in the commissioning and joining subtasks, accompanied with decreases in errors and cognitive loads. Such assistance is important for tasks with high complexity.

## Experiment 1: Effects of AR Assistance on the Performance of the Overall Assembly Task and the Two Subtasks

### Methods

#### Participants

Experiment 1 involved 47 students of Zhejiang Sci-Tech University. The AR-assisted group had 24 participants (12 females and 12 males) with a mean age of 20.63, whereas the screen-based documentation-assisted group had 23 participants (14 females and 9 males) with a mean age of 20.32.

#### Experimental Apparatus

**FIGURE 2:**
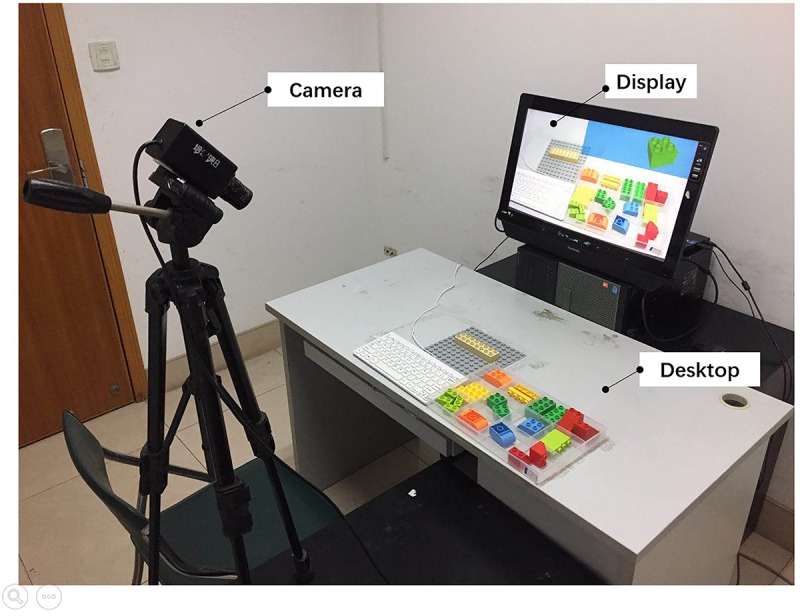
Experimental setup.

The entire experimental environment was composed of a desktop, display, and camera. Figure 2 shows the experimental setup.

The experimental program was developed by Unity3D, which can realize 3D video games and build visualizations and real-time 3D animation. This development tool is common in current applications of AR technology.

##### Desktop

The desktop is a tool that implements AR-assisted assembly tasks. The desktop was divided into the following zones:

(1) The assembly zone is the region for assembly operation. This zone was fixed with a piece of 19 cm × 19 cm baseboard of a Duplo series of Lego building blocks. In all experiments, the assembly task should be accomplished on the fixed position of the baseboard.
(2) The control zone is the area for the keyboard used to control the program. The start, progress, and end of the experiment were controlled by the space key on the keyboard.
(3) The alternative part zone is the area for essential alternative parts in the assembly task. A 4 × 3 12-grid box was used. In all experiments, the participants were asked to search the target parts from this box.

##### Display

A 19^″^ LCD with a resolution of 1920 × 1080 was used in the experiment.

In the beginning of the assembly task, the participants could see the entire desktop on the display so that they could focus on the AR information and the current task from the display. This design could relieve mental workload and reduce visual shifts (Wang and Dunston, 2006a).

All images in the assembly guideline were modeled and rendered using SketchUp 2018 and were drawn into images with a perspective angle. These images were displayed on the top right corner (blank area on the desktop) of the screen. Under non-AR assistance, this design restored the process of visual shifting between different regions.

**FIGURE 3:**
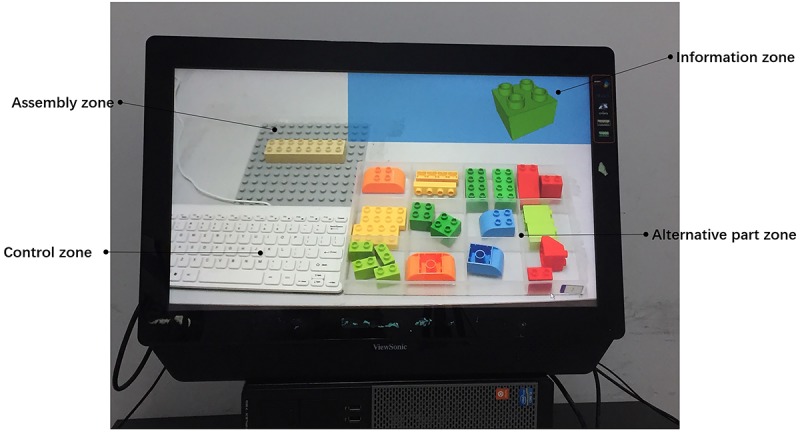
Zoning on display.

The pictures on the display reflected the entire desktop situation. The display was divided into information, assembly, control, and alternative part zones in accordance with the zoning of the desktop (Figure 3).

##### Camera

In the experiments, a 1080P high-resolution conference camera, which could ensure sharpness of pictures, was used. The visual angle of the pictures captured by the camera was similar to that of the images observed by the participants. The 19^″^ LCD displayed the pictures captured by the camera on the screen, and the AR assembly instruction was superimposed at the particular location of the screen in real time. Thus, the position and height of the camera were fixed to prevent the movement of the instruction relative to the building blocks.

#### Experimental Materials

**FIGURE 4:**
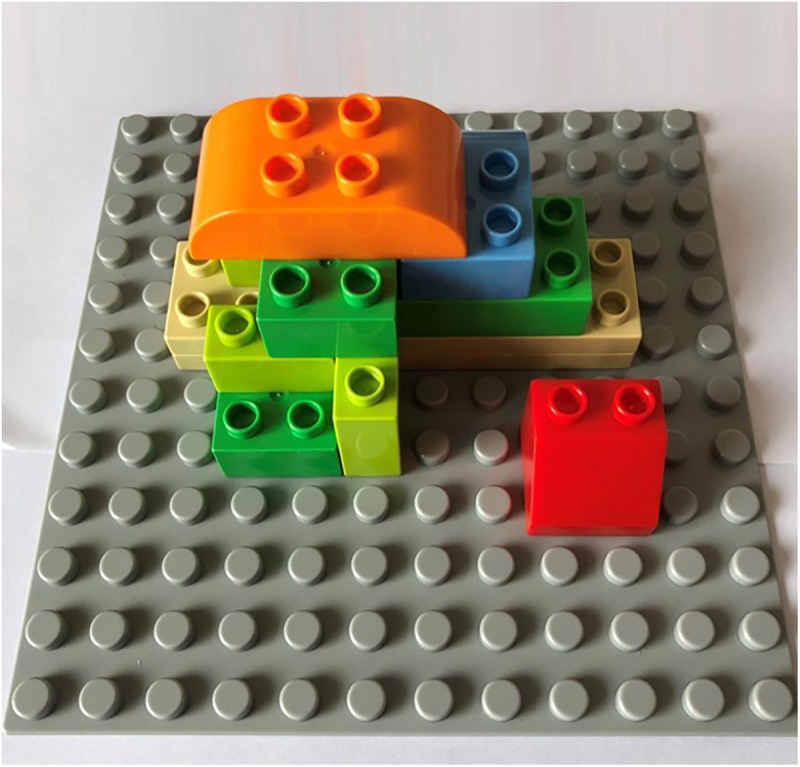
Final assembled solid figure in Experiment 1.

A Duplo series of large-grain building blocks of Lego was used in Experiment 1. Richardson et al. (2004) found that the number of selections, components, and assembly steps significantly impacted the complexity of the assembly task. In addition, the preliminary experiment determined that when the number of building parts was less than five, a ceiling effect occurred. Hence, to prevent the ceiling effect, participants were asked to assemble a solid figure using nine building blocks (components). Twelve parts with different sizes and shapes (selections) were available in the alternative part zone. Among them, only seven were designated as target parts, which were used in the assembly task. In addition, each target had one interferential part of the same color and similar shape. A total of nine steps were required to complete this assembly task (assembly steps). Figure 4 shows the final assembled solid figure.

#### Experimental Design

Experiment 1 used the between-subject design. The assembly assistance mode (screen-based documentation or AR) was the independent variable. Dependent variables included time of the overall assembly task, time of commissioning subtask, time of joining subtask, total mistakes, mistakes in commissioning subtask, mistakes in joining subtask, and ratings of cognitive loads (including the psychological effort and task difficulty dimensions).

#### Experimental Process

Experiment 1 covered three stages.

(1) Pretest: Purdue spatial visualization test.

The pretest was administered to test the intrinsic spatial cognitive ability of each participant and to classify them into groups on the basis of the results to reduce experimental errors. The participants’ mental rotation ability was evaluated by the Purdue spatial visualization test (rotation test) (Guay, 1980). The participants were divided into two groups on the basis of the test results, considering the balanced spatial cognitive ability between the groups. The average score of the AR-assisted group was 11.46 (*SD* = 2.978) whereas that of the screen-based documentation-assisted group was 10.87 (*SD* = 2.528). No significant difference was observed between the groups [*t*_(45)_ = 0.729, *p* = 0.47].

(2) Formal experiment: The influence of AR on the performance of the overall assembly task and subtasks compared with screen-based documentation-assisted assembly was investigated in the experiment.

**FIGURE 5:**
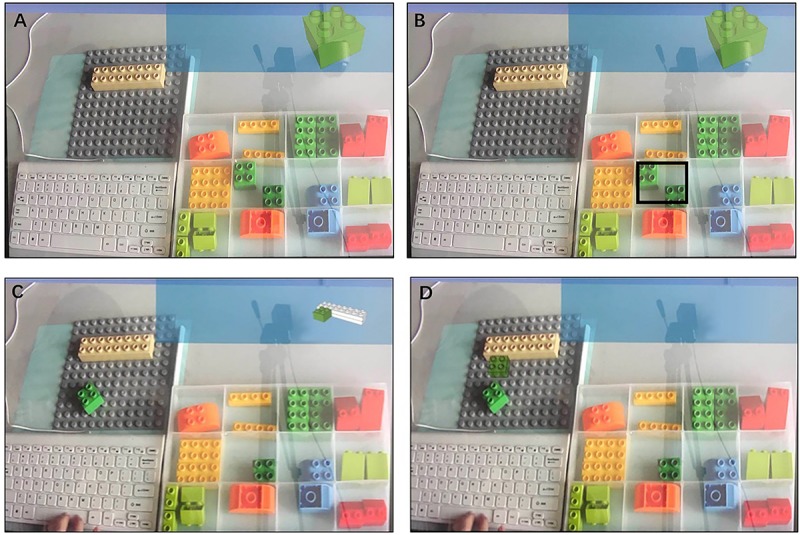
**(A)** Display of screen-based documentation-assisted group in commissioning subtask, **(B)** Display of AR-assisted group in commissioning subtask, **(C)** Display of screen-based documentation-assisted- assisted group in joining subtask, and **(D)** Display of AR-assisted group in joining subtask.

Under AR conditions, the picture of the target part was shown on the top right corner of the screen (information zone) when the participants clicked the space key. Moreover, the position of the target part in the alternative part zone was highlighted. The participants could recognize the target part by highlighting directly (Figure 5B). The participants read the assembly instruction of the target part, which was simultaneously displayed on the real-time images on the assembly zone, thereby allowing the participants to see the virtual image and real part concurrently (Figure 5D).

Given the screen-based documentation-assisted assembly, all image information was displayed on the top right corner of the screen (information zone) when the participants clicked the space key. No hint was provided on the alternative part and assembly zones; hence, participants must shift sights between zones (Figures 5A,C).

The participants clicked the space key, looked at the pictures of the target parts at the start of each assembly step, and captured the recognized target part from the alternative part zone. Then, the participants clicked the space key again to read the assembly instruction of the current step and place the building blocks after completely comprehending the assembly relationship. Subsequently, they clicked the space key to start the next step. This process was repeated until the nine steps were completed.

Participants controlled the experiment using the space key. The time point for the first click of the space key was recorded as P1, whereas the time points for the second and third clicks of the space key were recorded as P2 and P3. P2-P1 represented the time for the commissioning subtask, and P3-P2 represented the time for the joining subtask. Prior to the formal experiment, the participants were asked to practice once. The parts used in the practice were completely different from those in the formal experiment.

(3) Post-test: subjective evaluation of the cognitive load of PAAS.

A post-test was performed after the formal experiment to conduct a subjective evaluation on the cognitive load. The PAAS cognitive load subjective ratings were used (Paas and Van Merrienboer, 1994). The PAAS scale covers two dimensions: psychological effort and task difficulty. Many researchers appreciate this scale for its simplicity, convenience, and practicability. The evaluation of psychological efforts is sensitive to cognitive load, whereas the evaluation of task difficulty is sensitive to relevant cognitive loads (DeLeeuw and Mayer, 2008). This subjective evaluation has been proven to be highly valid for cognitive load evaluation (Ayres, 2006).

### Experimental Results

Data analysis results based on SPSS 21 are shown in Table 1.

**TABLE 1:** Differences of the two assistance modes in terms of the performance and cognitive load of the assembly task.

**Index**	**Screen-based documentation-assisted group (*n* = 23)**	**AR-assisted group (*n* = 24)**	***t***
Total time (s)	159.641±34.512	131.432±36.761	2.710^∗∗^
The time for the commissioning subtask (s)	56.143±11.569	46.399±13.561	2.645^*^
The time for the joining subtask (s)	103.498±29.784	85.033±26.191	2.260^*^
Total mistakes	1.780±1.043	0.290±0.624	5.978^∗∗∗^
Mistakes in the commissioning subtask	0.830±0.650	0.080±0.282	5.117^∗∗∗^
Mistakes in the joining subtask	0.960±0.638	0.210±0.415	4.786^∗∗∗^
Ratings of psychological effort	5.520±1.831	5.500±1.588	0.044
Ratings of task difficulty	4.480±2.042	3.830±1.786	1.154

^*^p < 0.05, ^∗∗^*p* < 0.01, ^∗∗∗^*p* < 0.001.

According to the independent *t*-test,

(1) Significant differences on the time of overall assembly task between the two groups [*t*_(45)_ = 2.710, *p* < 0.01] were observed. The average times for the commissioning and joining subtasks of the AR-assisted group were significantly shorter than those of the screen-based documentation-assisted group [*t*_(45)_ = 2.645, *p* < 0.05; *t*_(45)_ = 2.260, *p* < 0.05].
(2) Significant differences were observed in terms of total mistakes between the two groups [t_(45)_ = 5.978, *p* < 0.001]. The AR-assisted group showed significantly fewer mistakes in the commissioning and joining subtasks compared with the screen-based documentation-assisted group [*t*_(45)_ = 5.117, *p* < 0.001; *t*_(45)_ = 4.786, *p* < 0.001].
(3) With respect to the subjective evaluation of the participants of the task, the AR-assisted group showed slightly lower scores on psychological effort and task difficulty compared with the screen-based documentation-assisted group, but no significant difference was found.

Experiment 1 proves that AR assistance is conducive to shortening the overall assembly time and reducing mistakes during assembly. Moreover, it can significantly improve the performance of the commissioning and joining subtasks. However, it does not significantly affect cognitive loads. The stages of the assembly task in which the AR significantly works will be investigated in Experiments 2 and 3.

## Experiment 2: Effects of AR Assistance on the Performance of the Commissioning Subtask in Different Stages

### Methods

#### Participants

Eighty-nine participants, which include the students of Zhejiang Sci-Tech University, were invited. The AR-assisted group had 47 participants (25 females and 22 males) with a mean age of 20.49, whereas the screen-based documentation-assisted group had 42 participants (22 females and 20 males) with a mean age of 20.41.

#### Experimental Apparatus

The experimental apparatuses were the same as those in Experiment 1.

#### Experimental Materials

**FIGURE 6:**
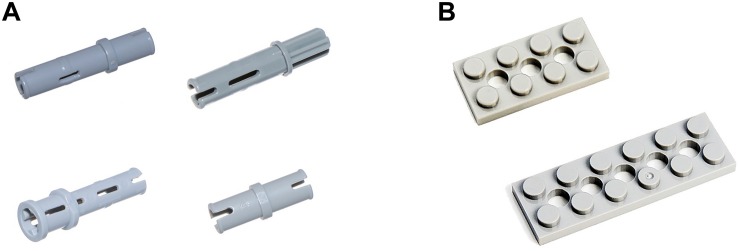
Assembly tasks of different complexities. **(A)** High-complexity task with four alternative parts. **(B)** Low-complexity task with two alternative parts.

The Technic small-grain series of Lego building blocks were used in Experiment 2. The alternative part zone had 12 parts with different shapes and sizes. All parts were gray. Duncan and Humphreys (1989) found that for all materials, complexity of visual search increased with the increased similarity of targets to non-targets. Moreover, Desimone and Duncan (1995) stated that the number of non-targets that were similar to the target greatly affected the complexity of the visual search task. Thus, on the basis of the complexity of the assembly task, each target part had one or three interferential parts with similar shapes. In the low-complexity task, each target part had one interference part with a similar shape (Figure 6B). In the high-complexity task, each target part had three interference parts with a similar shape. Thus, more time and resources were required for participants to recognize the correct part. Therefore, the participants were likely to select the wrong part (Figure 6A).

#### Experimental Design

Experiment 2 used the between-subject design. The assembly assistance mode (screen-based documentation or AR) and task complexity (high or low) were the independent variables. Dependent variables included time of perception and response selection, time of action, number of mistakes, and cognitive load ratings (including the psychological effort and task difficulty dimensions).

#### Experimental Process

(1) Pretest: The procedure was identical to that in Experiment 1. The average score of the AR-assisted group with a high-complexity task, the AR-assisted group with a low-complexity task, the screen-based documentation-assisted group with a high-complexity task, and the screen-based documentation-assisted group with a low-complexity task were 11.00 (*SD* = 2.65), 11.43 (*SD* = 2.56), 10.73 (*SD* = 2.99), and 10.70 (*SD* = 3.29), respectively. In addition, no significant difference was observed among the four groups [*F*_(3,85)_ = 0.313, *p* = 0.816].
(2) Formal experiment: The influences of AR-assisted assembly on the performance of the commissioning subtask with various complexities in different stages compared with screen-based documentation-assisted assembly were investigated.

**FIGURE 7:**
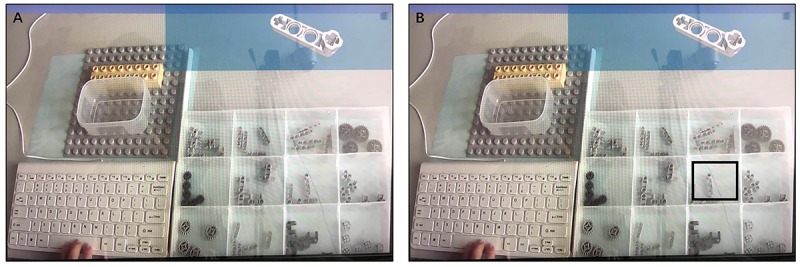
**(A)** Displays under screen-based documentation-assisted assembly task and **(B)** Display under AR-assisted assembly task.

Under AR-assisted assembly, an image of the target parts was shown on the top right corner of the screen (information zone) when the participants pressed the space key. Moreover, the position of the target part in the alternative part zone was highlighted. The participants could recognize the target part by highlighting directly (Figure 7B).

Given the screen-based documentation-assisted assembly, an image of the target parts was displayed on the top right corner of the screen (information zone) when the participants clicked the space key. No hint was provided on the alternative part zone, and the participants must shift sights between zones (Figure 7A).

The participants must look at the pictures of the target parts, recognize the accurate part from the alternative part zone, and capture it into the front box. Each participant performed 16 trials.

The participants controlled the experimental process using the space key on the keyboard. The time point for the first click of the space key was recorded as P1. After recognizing the target part in the alternative part zone, the time point at releasing the space key and capturing the target part was recorded as R1. The time point for the second click of the space key was recorded as P2. R1-P1 represented the time of perception and response selection, and P2-R1 represented the time of action. Four trials were performed prior to the formal experiment. The parts used in these trials were completely different from those in the formal experiment.

(3) Post-test: The same as that in Experiment 1.

### Experimental Results

Data analysis results based on SPSS 21 are shown in Table 2.

**TABLE 2:** Performance data of the commissioning subtask with various complexities in different stages under two assistance modes.

**Index**	**Screen-based documentation -assisted group**		**AR-assisted group**		***F***
		
	**Low complexity (*n* = 23)**	**High complexity (*n* = 24)**	**Low complexity (*n* = 23)**	**High complexity (*n* = 24)**	
Time of perception and response selection (s)	92.434±36.614	117.257±32.302	18.779±7.854	22.764±9.229	*F*_*m*_ = 262.475^∗∗∗^ *F*_*c*_ = 7.704^∗∗^ *F_*m*__×__*c*_* = 4.031^*^
Time of action (s)	61.726±20.543	64.525±30.054	48.810±13.993	51.035±14.345	*F*_*m*_ = 9.109^∗∗^ *F*_*c*_ = 0.330 *F_*m*__×__*c*_* = 0.004
Number of mistakes	1.700±1.593	3.270±1.804	0.130±0.626	0.170±0.381	*F*_*m*_ = 80.068^∗∗∗^ *F_*c*_* = 9.481^∗∗^ *F_*m*__×__*c*_* = 8.647^∗∗^
Ratings of psychological effort	6.150±1.182	6.640±1.706	4.830±1.969	5.420±1.586	*F*_*m*_ = 13.195^∗∗∗^ *F_*c*_* = 2.365 *F_*m*__×__*c*_* = 0.022
Ratings of task difficulty	5.100±1.334	5.450±1.792	2.870±1.359	2.920±1.558	*F*_*m*_ = 54.129^∗∗∗^ *F_*c*_* = 0.384 *F_*m*__×__*c*_* = 0.225

^*^p < 0.05, ^∗∗^*p* < 0.01, ^∗∗∗^*p* < 0.001. *F*_*m*_ indicates the *F*-value of the main effect of assistance modes. *F*_*c*_ indicates the *F*-value of the main effect of complexity. *F_*m*__×__*c*_* indicates the *F*-value of the interaction effect of assistance modes and complexity.

According to ANOVA, the main effect of the assistance mode (*Wilks*λ = 0.145, *p* < 0.001, $\eta_{p}^{2}$ = 0.855), the main effect of task complexity (*Wilks*λ = 0.771, *p* < 0.01, $\eta_{p}^{2}$ = 0.229), and the interaction effect of assistance mode and task complexity (*Wilks*λ = 0.831, *p* < 0.05, $\eta_{p}^{2}$ = 0.169) were all significant. This finding implied that assistance mode and task complexity influenced the dependent variables significantly. Moreover, the interaction of assistance mode and task complexity influenced the dependent variables remarkably. In other words, the influences of assistance mode on the dependent variables varied by task complexity.

#### Time of Perception and Response Selection

**FIGURE 8:**
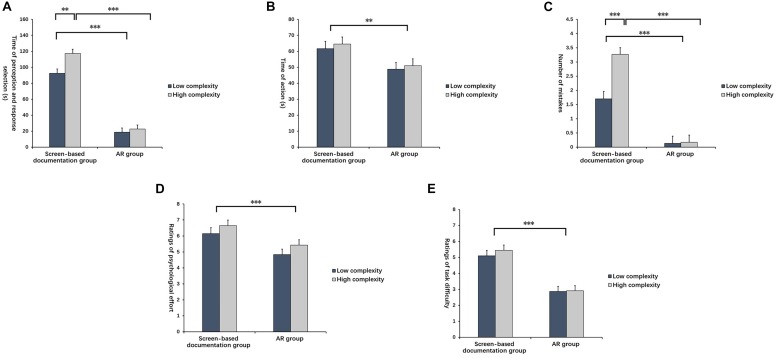
Indexes in commissioning subtask with various complexities under two assistance modes. **(A)** Time of perception and response selection, **(B)** Time of action, **(C)** Number of mistakes, **(D)** Ratings of psychological effort, and **(E)** Ratings of task difficulty. ^∗∗^Indicates *p* < 0.01; ^∗∗∗^indicates *p* < 0.001.

ANOVA revealed that the main effect of assistance mode on perception and response selection was significant [*F*_(1,85)_ = 262.475, *p* < 0.001, $\eta_{p}^{2}$ = 0.755]. According to the *post hoc* test, the average time of AR assistance was significantly shorter than that of screen-based documentation assistance (*p* < 0.001). The main effect of task complexity was also significant [*F*_(1,85)_ = 7.704, *p* < 0.01, $\eta_{p}^{2}$ = 0.083]. The *post hoc* test revealed that the average time of the low-complexity task was dramatically shorter than that of the high-complexity tasks (*p* < 0.01). The interaction of assistance mode and task complexity had a significant effect on the time of perception and response selection [*F*_(1,85)_ = 4.031, *p* < 0.05, $\eta_{p}^{2}$ = 0.045]. Simple effect analysis showed that under AR assistance, the times of the high and low complexity tasks had no differences (*p* > 0.05). However, the time of the high-complexity task under screen-based documentation assistance was significantly longer than that that of the low-complexity task (*p* < 0.01) (see Figure 8A).

#### Time of Action

According to ANOVA, the main effect of assistance mode was significant in the action stage [*F*_(1,85)_ = 9.109, *p* < 0.01, $\eta_{p}^{2}$ = 0.097]. The *post hoc* test pointed out that the average time of action under AR assistance was significantly shorter than that under screen-based documentation assistance (*p* < 0.01). No other significant effects were observed (see Figure 8B).

#### Number of Mistakes

ANOVA revealed that assistance mode influenced the number of mistakes significantly [*F*_(1,85)_ = 80.068, *p* < 0.001, $\eta_{p}^{2}$ = 0.485]. According to the *post hoc* test results, the number of mistakes under AR assistance was significantly lower than that under screen-based documentation assistance (*p* < 0.001). The main effect of task complexity was also significant [*F*_(1,85)_ = 9.481, *p* < 0.01, $\eta_{p}^{2}$ = 0.100]. The *post hoc* test showed that the number of mistakes in the low-complexity task was significantly lower than that in the high-complexity task (*p* < 0.01). The interaction effect of assistance mode and task complexity was significant [*F*_(1,85)_ = 8.647, *p* < 0.01, $\eta_{p}^{2}$ = 0.092]. According to the simple effect analysis, no significant effect was observed in the number of mistakes between the high and low complexity tasks under AR assistance. However, the number of mistakes in the high-complexity task under screen-based documentation assistance was significantly higher than that in the low-complexity task (*p* < 0.001) (see Figure 8C).

#### Ratings of Psychological Effort

According to ANOVA, the main effect of assistance mode was significant [*F*_(1,85)_ = 13.195, *p* < 0.001, $\eta_{p}^{2}$ = 0.134]. The *post hoc* test demonstrated that the ratings of psychological effort under AR assistance was significantly lower than that under screen-based documentation assistance (*p* < 0.001). No other significant effects were observed (see Figure 8D).

#### Ratings of Task Difficulty

According to ANOVA, the main effect of assistance mode was significant [*F*_(1,85)_ = 54.129, *p* < 0.001, $\eta_{p}^{2}$ = 0.389]. The *post hoc* test demonstrated that the rating of task difficulty under AR assistance was significantly lower than that under screen-based documentation assistance (*p* < 0.001). No other significant effects were observed (see Figure 8E).

## Experiment 3: Effects of AR Assistance on the Performance of Joining Subtask in Different Stages

### Methods

#### Participants

The participants in this experiment were the same as those in Experiment 2. To avoid the order effect, the sequences of the two experiments were counter-balanced. Half of the participants conducted Experiment 2 firstly, and the other half conducted Experiment 3 firstly. In addition, to decrease the learning effect, for each participant, the setup of the assistance mode and task complexity was balanced between Experiments 2 and 3. For example, if a participant performed the high-complexity task with AR assistance in Experiment 2, then he/she would perform the low-complexity task with screen-based documentation assistance in Experiment 3.

#### Experimental Apparatus

Experimental apparatuses were the same as those in Experiment 1.

#### Experimental Materials

A Duplo series of large-grain building blocks of Lego was used in Experiment 3. The participants were asked to assemble solid figures using nine pieces of building blocks.

**FIGURE 9:**
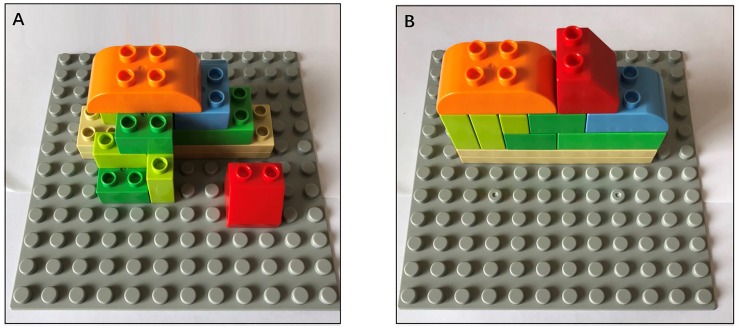
Tasks with various complexities. **(A)** High-complexity task, **(B)** Low-complexity task.

The complexity of the task depends on novel assemblies and directions of the solid figure of target building. Figure 9A shows the final assembled solid figure in the high-complexity task. Three novel building blocks were included in the high-complexity task [according to Richardson et al. (2004), the factor “novel assemblies” influences the complexity of the assembly task]. One of the three novel building blocks was required to secure the parts separated from the main building body. The other two novel building blocks were not required to secure all the fastenings. By contrast, all other building blocks are required to secure all the fastenings. In addition, the solid figure in the high-complexity task was extended to different directions. Hence, the participants should continuously perform mental rotation to finish the building, thereby increasing their cognitive loads and the complexity of the task (Shepard and Metzler, 1971; Tang et al., 2003). This setup was identical to that in Experiment 1.

In the low-complexity task, the number of building blocks and assembly steps was the same as that in the high-complexity task. However, the assembly task in this condition had no novel assemblies. Moreover, the solid figure was extended to the same direction. Hence, the participants easily judged and even predicted the building position, with a low possibility of committing errors. Figure 9B shows the final assembled solid figure in the low-complexity task.

#### Experimental Design

Experiment 3 used the between-subject design. The assembly assistance mode (screen-based documentation or AR) and task complexity (high or low) were the independent variables. Dependent variables included time of perception and response selection, time of action, number of mistakes, and cognitive load ratings (including the psychological effort dimension and task difficulty dimension).

#### Experimental Process

(1) Pretest: The same as that in Experiment 1.
(2) Formal experiment: The influences of AR-assisted assembly on the performance of joining subtask with various complexities in different stages compared with screen-based documentation-assisted assembly were investigated in the experiment.

**FIGURE 10:**
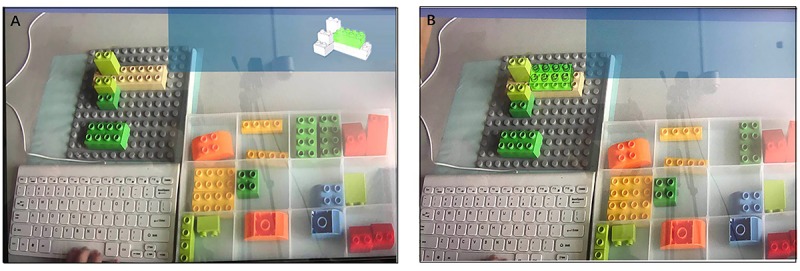
**(A)** Displays under screen-based documentation-assisted assembly task, **(B)** Display under AR-assisted assembly task.

Under the AR-assisted assembly, the assembly instruction of the current step was shown on the real-time image of the assembly zone, which was captured by the camera when the participants pressed the space key. The participants could simultaneously view the virtual images and real parts (Figure 10B).

Given the screen-based documentation-assisted assembly, the assembly instruction of the current step was displayed on the top right corner of the screen (information zone) when the participants clicked the space key. No hint was provided on the assembly zone, and the participants must understand the building position and relations of the target parts. In this process, the participants must make visual shifts between regions (Figure 10A).

Nine completely the same parts were applied in the tasks with different complexities.

At the beginning of the experiment, the participants clicked the space key, and the pictures of the target parts were displayed on the information zone. The position of the target part in the alternative part zone was highlighted simultaneously on the AR-assisted condition. After recognizing the target part, the participants released and captured the space key from the alternative part zone. This process was defined as the commissioning subtask and was not analyzed in this experiment. Then, the participants clicked the space key again and read the assembly instruction of the current step. The time point for the second click of the space key was recorded as P1. After the assembly instruction of the current step was understood, the time point at releasing the space key to build blocks was recorded as R1. After assembling the current step, the participants clicked the space key for the third time to view the picture of the next target part, at which a new circulation started. The time point at clicking the space key for the third time was recorded as P2. R1-P1 represented the time of perception and response selection, and P2-R1 represented the time of action. This process was repeated in all nine steps. One trial was performed prior to the formal experiment. The parts used in this trial were completely different from those in the formal experiment.

(3) Post-test: The same as that in Experiment 1.

### Experimental Results

Data analysis results based on SPSS 21 are shown in Table 3.

**TABLE 3:** Performance data of the joining subtask with various complexities in different stages under two assistance modes.

**Index**	**Screen-based documentation-assisted group**		**AR-assisted group**		***F***
		
	**Low complexity (*n* = 23)**	**High complexity (*n* = 24)**	**Low complexity (*n* = 23)**	**High complexity (*n* = 24)**	
Time of perception and response selection (s)	17.357±8.766	52.316±20.476	11.923±3.522	31.581±12.814	*F*_*m*_ = 22.517^∗∗∗^ *F*_*c*_ = 98.084^∗∗∗^ *F_*m*__×__*c*_* = 7.697^∗∗^
Time of action (s)	46.274±16.085	64.166±19.435	46.820±15.922	66.825±23.216	*F*_*m*_ = 0.157 *F*_*c*_ = 21.931^∗∗∗^ *F_*m*__×__*c*_* = 0.068
Number of mistakes	0.200±0.894	1.000±0.926	0.170±0.650	0.170±0.381	*F*_*m*_ = 7.584^∗∗^ *F_*c*_* = 6.453^*^ F_*m*__×__*c*_ = 6.691^*^
Ratings of psychological effort	5.200±1.361	6.090±1.571	5.350±2.058	6.080±1.717	*F*_*m*_ = 0.037 *F*_*c*_ = 5.019^*^ F_*m*__×__*c*_ = 0.046
Ratings of task difficulty	3.800±1.576	4.410±2.153	3.480±1.310	4.250±1.648	*F*_*m*_ = 0.445 *F*_*c*_ = 3.666 *F_*m*__×__*c*_* = 0.051

^*^p < 0.05, ^∗∗^*p* < 0.01, ^∗∗∗^*p* < 0.001. *F*_*m*_ indicates the *F*-value of the main effect of assistance modes. *F*_*c*_ indicates the *F*-value of the main effect of complexity. *F_*m*__×__*c*_* indicates the *F*-value of the interaction effect of assistance modes and complexity.

According to ANOVA, the main effect of assistance mode (*Wilks* λ = 0.691, *p* < 0.001, $\eta_{p}^{2}$ = 0.309), the main effect of task complexity (*Wilks*λ = 0.434, *p* < 0.001, $\eta_{p}^{2}$ = 0.566), and the interaction effect of assistance mode and task complexity (*Wilks*λ = 0.816, *p* < 0.01, $\eta_{p}^{2}$ = 0.184) were significant. This finding implied that assistance mode and task complexity influenced the dependent variables significantly. Moreover, the interaction of assistance mode and task complexity influenced the dependent variables remarkably. In other words, the influences of assistance mode on the dependent variables varied by task complexity.

#### Time of Perception and Response Selection

**FIGURE 11:**
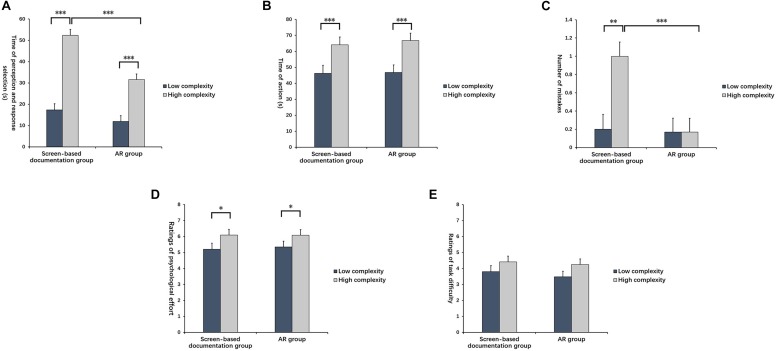
Indexes in joining subtask with various complexities under two assistance modes. **(A)** Time of perception and response selection, **(B)** Time of action, **(C)** Number of mistakes, **(D)** Ratings of psychological effort, and **(E)** Ratings of task difficulty. ^*^Indicates *p* < 0.05; ^∗∗^indicates *p* < 0.01; ^∗∗∗^indicates *p* < 0.001.

ANOVA revealed that the main effect of assistance mode on perception and response selection was significant [*F*_(1,85)_ = 22.517, *p* < 0.001, $\eta_{p}^{2}$ = 0.209]. According to the *post hoc* test, the average time of perception and response selection under AR assistance was significantly shorter than that under screen-based documentation assistance(*p* < 0.001). The main effect of task complexity was significant [*F*_(1,85)_ = 98.084, *p* < 0.001, $\eta_{p}^{2}$ = 0.536]. The *post hoc* test revealed that the average time of the low-complexity task was dramatically shorter than that of the high-complexity task (*p* < 0.001). The interaction effect of assistance mode and task complexity was significant [*F*_(1,85)_ = 7.697, *p* < 0.01, $\eta_{p}^{2}$ = 0.083]. The simple effect analysis showed that the accomplishment time of the high-complexity task under AR assistance was significantly shorter than that under screen-based documentation assistance (*p* < 0.001). However, no significant difference was observed in the time of the low-complexity task between AR assistance and screen-based documentation assistance (see Figure 11A).

#### Time of Action

According to the ANOVA, the main effect of assistance mode was not significant [*F*_(1,85)_ = 0.157, *p* > 0.05, $\eta_{p}^{2}$ = 0.002]. The main effect of task complexity was significant [*F*_(1,85)_ = 21.931, *p* < 0.001, $\eta_{p}^{2}$ = 0.205]. The *post hoc* test pointed out that the average time of action in the low-complexity task was significantly shorter than that of the high-complexity task (*p* < 0.001). The interaction effect of assistance mode and task complexity was not significant (see Figure 11B).

#### Number of Mistakes

The main effect of assistance mode was significant [*F*_(1,85)_ = 7.584, *p* < 0.01, $\eta_{p}^{2}$ = 0.082]. According to *post hoc* test results, the number of mistakes under AR assistance was significantly lower than that under screen-based documentation assistance(*p* < 0.01). The main effect of task complexity was significant [*F*_(1,85)_ = 6.453, *p* < 0.05, $\eta_{p}^{2}$ = 0.071]. The *post hoc* test revealed that the number of mistakes in the low-complexity task was significantly lower than that in the high-complexity task (*p* < 0.05). The interaction of assistance mode and task complexity had a significant effect on the number of mistakes [*F*_(1,85)_ = 6.691, *p* < 0.05, $\eta_{p}^{2}$ = 0.073]. According to the simple effect analysis, no significant effect was observed on the number of mistakes between the high and low complexity tasks under AR assistance. However, the number of mistakes in the high-complexity task under screen-based documentation assistance was significantly higher than that in the low-complexity task (*p* < 0.01) (see Figure 11C).

#### Ratings of Psychological Effort

According to ANOVA, only the main effect of task complexity was significant [*F*_(1,85)_ = 5.019, *p* < 0.05, $\eta_{p}^{2}$ = 0.056]. The *post hoc* test demonstrated that the ratings of psychological effort in the low-complexity task was significantly lower than that in the high-complexity task (*p* < 0.05) (see Figure 11D).

#### Ratings of Task Difficulty

According to ANOVA, no main effect of assistance mode or task complexity nor the interaction effect between the two factors was observed on the ratings of task difficulties (see Figure 11E).

## Discussion

### Effects of AR Assistance on the Overall Assembly Task and Commissioning and Joining Subtasks

In Experiment 1, the AR assistance can shorten the time of the overall assembly task and commissioning and joining subtasks and can reduce errors in these tasks. This finding is consistent with most of the associated studies. AR integrates regions involved in the assembly task, thereby decreasing the cost of information access. Under screen-based documentation assistance, user attention must be shifted constantly in the information, assembly, and alternative part zones, during which head and eye movements consume considerable time and physical power. The cognitive shift between the information source and environment may also increase the time cost and errors (Fischer et al., 1980; Weintraub et al., 1985; Larish and Wickens, 1991). Under AR assistance, virtual information is overlapped on the real picture shot by the camera directly. Users only need to shift attention between the assembly and alternative part zones, thereby reducing time cost and errors. The PPSA scale results of the cognitive load in Experiment 1 reveal that the cognitive load perceived by the AR-assisted group is slightly lower than that of the screen-based documentation-assisted group. Such difference has no statistical significance. However, the time and error in the overall assembly task and subtasks reveal the main effect of assistance mode probably because the PAAS survey is not sufficiently sensitive to identify this difference. On the one hand, the validity of the PAAS might be insufficient to identify the difference between modes. On the other hand, the participants might fail to examine their cognitive process and thus fail to report the amount of mental effort expenditure (Paas and Van Merrienboer, 1994).

### Effects of AR Assistance on the Commissioning Subtask in Different Stages

In Experiment 2, AR assistance can reduce errors and cognitive loads of users in the commissioning subtask. In the perception and response selection stage of the commissioning subtask, AR assistance can shorten the time required to determine the target parts. Such assistance effect is crucial in tasks with high complexity. The position of the target parts is marked under AR conditions to guide the attention of the participants, thereby increasing the findability of the target part (Morville and Callender, 2010). Such exogenous stimulus drives an automatic and reflective shift of attention, thereby helping the users find the target part and effectively reduce search errors (Stork and Schubö, 2010) and cognitive loads. The AR system is the combination of the synthesized computer images and field of vision of users; it can attract user attention through arrows, tags, highlighted objects, and animations. The highlight used in this study is an effective approach for attracting user attention (Tang et al., 2003).

In the action stage of the commissioning subtask, AR assistance can shorten the time required to capture parts, which might be caused by the influences of information activity on working activity. In view of the attention demand level, the attention level for searching and understanding information is higher than that for workpiece operation (Neumann and Majoros, 1998). During visual searching based on AR assistance, the time required in the searching activity can be reduced. Instead, the user can focus on capturing the target part rapidly, thereby shortening the time of capture. Moreover, labeling the target part in the AR assistance can inhibit human attention on unrelated projects and facilitate the operators to start the movement early. In addition, the spatial clue provided by the AR assistance accelerates the entire movement probably due to the clues, which increases the confidence of the participants with their selections (Stork et al., 2008).

The task complexity in the commission subtask affects the perception and response selection stage but not the action stage. The reason may be that the manipulation of task complexity only influences the cognitive process in the commission subtask. When the participants identify which part to capture, no difference may exist in the subsequent activity stage between the low- and high-complexity conditions. In addition, regardless of the complexity of the commission subtask, the AR assistance can improve the assembly performance. This finding is different from that of Wiedenmaier et al. (2003), where the AR assistance only improved the assembly performance when the assembly task has high complexity. Wiedenmaier et al. (2003) did not distinguish the assembly task into different subtasks and stages and used an overall assembly task. In this approach, when the assembly task has low complexity, the absence of the effect of AR assistance in the joining subtask offsets the positive effect of the AR assistance in the commission subtask, thereby interpreting our results. Hence, Experiment 2 reveals that the AR assistance can improve the assembly task performance in the commission subtask even when the assembly task has low or high complexity.

The complexity of the task does not affect the cognitive load. However, the time of perception and response selection and the number of errors reveal the main effect of the task complexity. Hence, PAAS survey is insufficiently sensitive to identify this difference.

### Effects of AR Assistance on the Joining Subtask in Different Stages

Experiment 3 concludes that AR assistance can reduce the number of errors in the joining subtask, but it does not decrease the cognitive loads of users.

In the perception and response selection stage of the joining subtask, AR assistance can shorten the time required to understand the assembly relation, especially in the tasks with high complexity. In Experiment 3, the real object (physical part) that the users observed is displayed from the self-centered perspective, whereas the screen-based documentation assistance on the display is shown on the external center perspective. The users often rotate the object to the angle agreeing with the picture or attempt to map the media images onto the object in mind for the convenience of recognition. Such conversion increases the cognitive loads (Shepard and Metzler, 1971). In this experiment, AR helps the users effectively maintain the information and working activities from the self-centered perspective, reduce the process of psychological rotation, and shorten the task time.

In the action stage of the joining subtask, AR assistance fails to shorten the time for users to build parts in the high- and low-complexity tasks. On the contrary, it even prolongs the time for users to accomplish the action stage probably because cognitive time (for reading assembly map and understanding the assembly relation) is independent from handwork time (actual time for part building). Previous studies have indicated that individuals spend different times for cognitive or information activities, but the time engaged in handwork is nearly the same (Neumann and Majoros, 1998). Therefore, the performance difference of users under different task complexities and assistance modes is only reflected on cognitive time.

When the task complexity is low, no significant difference is found in the time of perception and response selection and the number of mistakes between assistance modes. One possible interpretation is that, in the low-complexity task, the participants are not required to expend abundant mental resources to match the visual angle, and they are less likely to commit errors. Therefore, the advantages of AR may not be reflected. However, a significant difference was observed when the task had high complexity. Thus, the AR assistance may only improve the assembly performance in joining subtask when the assembly task has high complexity. This finding is consistent with that of Wiedenmaier et al. (2003) probably because the participants are required to expend abundant psychological resources in the high-complexity task. Thus, the advantages of the AR assistance in reducing the psychological rotation could be reflected. Combined with the results in the commission subtask, this study validates the findings of Wiedenmaier et al. (2003) only in the joining subtask.

In addition, the PPSA scale results of the cognitive loads reflect that the ratings of the psychological effort of participants under AR assistance have insignificant difference from that under screen-based documentation assistance and are even increased in the low-complexity task. Nevertheless, many previous researchers have proven that if the operators are not required to transform the object in mind and maintain the relation model between the assembly object and its position in working memory, then the workload of the brain is decreased (Tang et al., 2003). However, this relationship is not proven by Experiment 3. On the one hand, the advantage of overlapping the augmented information on the building body is offset by the cost of visual interference (Tang et al., 2003). On the other hand, limited by technology, the augmented information does not completely conform to the physical parts in the experiment, thereby potentially decreasing the initial advantages of AR assistance (Khuong et al., 2014). For AR-assisted assembly, whether displaying the assembly commander by overlapping information is the most appropriate display mode still requires further discussion in future studies.

On the basis of these discussions, the relationship between assembly guideline and physical object might affect task performance. One core problem in the application of AR is combining real and virtual objects into an actual environment to enable users to interact with them simultaneously (Trevisan et al., 2002). In the assisted assembly system, the designers must consider the demands of the assembly task in terms of perception, cognition, and functions. The virtual information must be designed in accordance with the specific equipment and tasks (subtask and different stages of each subtask) to realize the continuity of human, computer, and environment.

## Conclusion

Augmented reality assistance can shorten the time of the overall assembly task and subtasks (commissioning and joining) and reduce errors during these tasks. Moreover, AR assistance can decrease cognitive load in the commissioning subtask, but it increases cognitive load in the joining task with low complexity.

In the perception and response selection stage of the commissioning and joining subtasks, AR assistance can shorten the time for users to recognize the target part and understand the assembly relation. This advantage is extremely significant for the task with high complexity. In the action stage of two subtasks, AR assistance can shorten the time for users to capture parts, but it prolongs the time to build parts.

In summary, the influences of AR assistance system on the performance of the overall assembly task and two subtasks are different. The influences of the AR assistance system on the performance of information and working activities are also different. Moreover, the effects of AR on the performance of the low- and high-complexity assembly tasks are different. For assembly tasks that satisfy the characteristics of Stork’s assembly task model, we suggest the following:

(1) The effects of the AR-assisted assembly system on the performance of subtasks should be evaluated to acquire further comprehensive information on system performance.
(2) The performance evaluation of different stages should be considered to optimize the AR-assisted assembly system. This action is conducive to focusing on the processes that must be optimized.
(3) When using AR in the information activity of the joining subtask, the guidelines should be displayed by placing the simulated virtual model beside the physical model considering the visual interference of augmented information to physical parts and sensitivity to dislocation, delay, and deep clue conflicts (Khuong et al., 2014).
(4) Augmented reality is not applicable to all assembly tasks. A series of studies on task performance and cognitive test should be performed prior to the development of an AR assistance system (Nee et al., 2012) to determine the type of tasks compatible with AR (Livingston, 2005).

### Limitation and Future Work

Due to technological limitations, the present study uses a desktop AR system, which relatively has differences from the real AR system. For example, the assembly visual angle is fixed in this study, but in the real AR system, the participants could turn their heads freely; thus, the range of vision is wider than that in this research. Moreover, the materials used in this study are building blocks, which may be different from the real assembly tasks. Fastening the building parts is simpler and the complexity is lower in this study than in the real assembly tasks in daily life. Therefore, using real assembly tasks to conduct experiments with the real AR system and setting additional levels of task complexity in the future are meaningful. In addition, only behavior data and the subjective questionnaire are used as dependent variables. Adding eye movement and interview data is necessary to probe further into the performance in the future work. For example, the glance duration on the AR instruction and real objects may interpret the mechanism of advantages of AR assistance. Investigating the effect of features and types of the AR graphics on the assembly performance is also interesting.

Although the research on AR technology has considerably progressed in the recent 20 years, the application of AR to the manufacturing industry remains in the exploration and prototype stages. AR assistance is integrated with intelligence development (Wang et al., 2016a). Future studies can develop intelligent AR assistance systems, which can provide commands that are appropriate for the cognitive process of the operator and in accordance with the operator’s psychological state. In the future, numerous functional modules related with user cognition will be integrated into the AR-assisted assembly systems. Therefore, attention should be paid to the compatibility and optimization of user cognition and technology integrated system (Wang et al., 2016b).

## Data Availability

All datasets generated for this study are included in the manuscript and/or the supplementary files.

## Ethics Statement

This study was carried out in accordance with the recommendations of “Human experiment ethics, Ethics committee of Zhejiang Sci-Tech University” with written informed consent from all subjects. All subjects gave written informed consent in accordance with the Declaration of Helsinki. The protocol was approved by the “Ethics committee of Zhejiang Sci-Tech University.”

## Author Contributions

HL and ZY designed the study and analyzed the data. JS and ZY wrote the manuscript. WJ, JS, YS, and YW participated in the participant recruitment and data collection. CK, SM, JS, and WJ participated in the data preprocessing and results discussion. HL supervised the whole study.

## Conflict of Interest Statement

The authors declare that the research was conducted in the absence of any commercial or financial relationships that could be construed as a potential conflict of interest.

## References

[B1] Ayres P. (2006). Using subjective measures to detect variations of intrinsic cognitive load within problems. *Learn. Instr.* 16 389–400. 10.1016/j.learninstruc.2006.09.001

[B2] Azuma R. Baillot Y. Behringer R. Feiner S. Julier S. MacIntyre B. (2001). Recent advances in augmented reality. *IEEE Comput. Graph. Appl.* 21 34–47.

[B3] Azuma R. T. (1997). A survey of augmented reality. Presence. *Teleoperators Virtual Enviro.* 6 355–385. 10.1162/pres.1997.6.4.355

[B4] Baird K. M. Barfield W. (1999). Evaluating the effectiveness of augmented reality displays for a manual assembly task. *Virtual Real.* 4 250–259. 10.1007/bf01421808

[B5] Boud A. C. Haniff D. J. Baber C. Steiner S. J. (1999). “Virtual reality and augmented reality as a training tool for assembly tasks,” in *1999 IEEE International Conference on Information Visualization*, (London).

[B6] Cheng K.-H. Tsai C.-C. (2012). Affordances of augmented reality in science learning: suggestions for future research. *J. Sci. Educ. Technol.* 22 449–462. 10.1007/s10956-012-9405-9

[B7] DeLeeuw K. E. Mayer R. E. (2008). A comparison of three measures of cognitive load: evidence for separable measures of intrinsic, extraneous, and germane load. *J. Educ. Psychol.* 100 223–234. 10.1037/0022-0663.100.1.223

[B8] Desimone R. Duncan J. (1995). Neural mechanisms of selective visual attention. *Annu. Rev. Neurosci.* 18 193–222. 10.1146/annurev.neuro.18.1.193 7605061

[B9] Duncan J. Humphreys G. W. (1989). Visual search and stimulus similarity. *Psychol. Rev.* 96:433 10.1037//0033-295x.96.3.433 2756067

[B10] Fischer E. Haines R. F. Price T. A. (1980). *Cognitive Issues in Head-up Displays.* Washington, D.C : NASA Tech.

[B11] Gavish N. Gutierrez T. Webel S. Rodriguez J. Tecchia F. (2011). “Design guidelines for the development of virtual reality and augmented reality training system for maintenance and assembly tasks,” in *Proceedings of BIOWeb of Conferences*, (EDP Sciences), 1.

[B12] Gorecky D. Worgan S. F. Strasse T. (2011). “COGNITO - A cognitive assistance and training system for manual tasks in industry,” in *European Conference on Cognitive Ergonomics*, (Rostock).

[B13] Guay R. B. (1980). “Spatial ability measurement: a critique and an alternative,” in *Paper presented at the Annual Meeting of the American Educational Research Association*, (Boston, MA).

[B14] Henderson S. J. Feiner S. K. (2011). “Augmented reality in the psychomotor phase of a procedural task,” in *2011 10th IEEE International Symposium on Mixed and Augmented Reality*, (Basel).

[B15] Hou L. Wang X. Bernold L. Love P. E. D. (2013). Using animated augmented reality to cognitively guide assembly. *J. Comput. Civil Eng.* 27 439–451. 10.1061/(asce)cp.1943-5487.0000184

[B16] Hou L. Wang X. Truijens M. (2015). Using augmented reality to facilitate piping assembly: an experiment-based evaluation. *J. Comput. Civil Eng.* 29:05014007 10.1061/(asce)cp.1943-5487.0000344

[B17] Johnson A. Proctor R. W. (2004). *Attention: Theory and Practice.* Thousand Oaks: Sage.

[B18] Khuong B. M. Kiyokawa K. Miller A. La Viola J. J. Mashita T. Takemura H. (2014). “The effectiveness of an AR-based context-aware assembly support system in object assembly,” in *Proceedings of IEEE virtual reality*, (Minneapolis), 57–62.

[B19] Lamberti F. Manuri F. Sanna A. Paravati G. Pezzolla P. Montuschi P. (2014). Challenges, opportunities, and future trends of emerging techniques for augmented reality-based maintenance. *IEEE Trans. Emerg. Top. Comput.* 2 411–421. 10.1109/tetc.2014.2368833

[B20] Larish I. Wickens C. D. (1991). “Attention and HUDs: Flying in the dark?,” in *Proceedings of the Society for Information Display*, (Playa del Rey, CA: Society of Information Display).

[B21] Leu M. C. ElMaraghy H. A. Nee A. Y. C. Ong S. K. Lanzetta M. Putz M. (2013). CAD model based virtual assembly simulation, planning and training. *CIRP Ann.* 62 799–822. 10.1016/j.cirp.2013.05.005

[B22] Livingston M. A. (2005). Evaluating human factors in augmented reality systems. *IEEE Comput. Graph. Appl.* 25 6–9. 10.1109/mcg.2005.130 16315470

[B23] Morville P. Callender J. (2010). *Search Patterns*, 1st Edn Sebastopol: O’Reilly.

[B24] Nee A. Y. C. Ong S. K. Chryssolouris G. Mourtzis D. (2012). Augmented reality applications in design and manufacturing. *CIRP Ann.* 61 657–679. 10.1016/j.cirp.2012.05.010

[B25] Neumann U. Majoros A. (1998). “Cognitive, performance, and systems issues for augmented reality applications in manufacturing and maintenance,” in *Proceedings of the IEEE Virtual Reality Annual International Symposium*, (Atlanta).

[B26] Nilsson S. Johansson B. (2007). “). Fun and usable: augmented reality instructions in a hospital setting,” in *Proceedings of the 19th Australasian conference on Computer-Human Interaction: Entertaining user interfaces*, (Adelaide, SA), 123–130.

[B27] Paas F. G. W. C. Van Merrienboer J. J. G. (1994). Instructional control of cognitive load in the training of complex cognitive tasks. *Educ. Psychol. Rev.* 6 351–371. 10.5014/ajot.2013.008078 23968802

[B28] Radkowski R. Herrema J. Oliver J. (2015). AR-based manual assembly support with visual features for different degrees of difficulty. *Int. J. Hum. Comput. Int.* 31 337–349. 10.1080/10447318.2014.994194

[B29] Richardson M. Jones G. Torrance M. (2004). Identifying the task variables that influence perceived object assembly complexity. *Ergonomics* 47 945–964. 10.1080/00140130410001686339 15204272

[B30] Sabine W. Uli B. Timo E. Matteo P. (2013). Augmented reality training for assembly and maintenance skills. *Rob. Auton. Syst.* 61 398–403. 10.1016/j.robot.2012.09.013

[B31] Seok K.-H. Kim Y. S. (2008). “A Study on Providing Prompt Assembly Information Using AR Manual,” in *2008 Third International Conference on Convergence and Hybrid Information Technology*, (Busan.).

[B32] Shepard R. N. Metzler J. (1971). Mental rotation of three-dimensional objects. *Science* 171 701–703. 10.1126/science.171.3972.701 5540314

[B33] Siltanen S. Hakkarainen M. Korkalo O. Salonen T. Saaski J. Woodward C. (2007). “Multimodal User Interface for Augmented Assembly,” in *2007 IEEE 9th Workshop on Multimedia Signal Processing*, (Chania).

[B34] Stoessel C. Wiesbeck M. Stork S. Zaeh H. Schuboe A. (2008). “Towards Optimal Worker Assistance: Investigating Cognitive Processes in Manual Assembly,” in *Manufacturing Systems and Technologies for the New Frontier*, eds Mitsuishi M. Ueda K. Kimura F. (London: Springer), 245–250. 10.1007/978-1-84800-267-8_50

[B35] Stork S. Hild I. Wiesbeck M. Zaeh M. F. Schubö A. (2009). “Finding relevant items: attentional guidance improves visual selection processes,” in *Proceedings of the HCI and Usability for e-Inclusion. USAB 2009. Lecture Notes in Computer Science*. eds Holzinger A. Miesenberger K. Berlin: Springer, 69–80. 10.1007/978-3-642-10308-7_5

[B36] Stork S. Schubö A. (2010). Human cognition in manual assembly: theories and applications. *Adv. Eng. Inform.* 24 320–328. 10.1016/j.aei.2010.05.010

[B37] Stork S. Stößel C. Schubö A. (2008). *The influence of instruction mode on reaching movements during manual assembly*. *Proceedings of the HCI and Usability for e-Inclusion. USAB 2008. Lecture Notes in Computer Science*. ed Holzinger A. Berlin: Springer, 161–172.

[B38] Tang A. Owen C. Biocca F. Mou W. (2003). “Comparative effectiveness of augmented reality in object assembly,” in *Proceedings of the Conference on Human Factors in Computing Systems*, (Fort Lauderdale,).

[B39] Trevisan D. Vanderdonckt J. Macq B. (2002). “Analyzing interaction in augmented reality systems,” in *Proceedings of ACM Multimedia’2002 International Workshop on Immersive Telepresence ITP*, (New York, NY), 56–59.

[B40] Wang X. Dunston P. S. (2006a). Compatibility issues in augmented reality systems for aec: an experimental prototype study. *Autom. Constr.* 15 314–326. 10.1016/j.autcon.2005.06.002

[B41] Wang X. Dunston P. S. (2006b). Potential of augmented reality as an assistant viewer for computer-aided drawing. *J. Comput. Civil Eng.* 20 437–441. 10.1061/(asce)0887-3801(2006)20:6(437)

[B42] Wang X. Ong S. K. Nee A. Y. C. (2016a). A comprehensive survey of augmented reality assembly research. *Adv. Manuf.* 4 1–22. 10.1007/s40436-015-0131-4

[B43] Wang X. Ong S. K. Nee A. Y. C. (2016b). Multi-modal augmented-reality assembly guidance based on bare-hand interface. *Adv. Eng. Inform.* 30 406–421. 10.1016/j.aei.2016.05.004

[B44] Webel S. Bockholt U. Engelke T. Gavish N. Tecchia F. (2011a). “Design recommendations for augmented reality based training of maintenance skills,” in *Recent trends of Mobile Collaborative Augmented Reality Systems*, eds Alem L. Huang W. (Berlin, Germany: Springer), 61–74.

[B45] Webel S. Bockholt U. Keil J. (2011b). “Design criteria for AR-based training of maintenance and assembly tasks,” in *Proceedings of the 2011 International Conference on Virtual and Mixed Reality*, (Orlando), 123–132. 10.1007/978-3-642-22021-0_15

[B46] Weintraub D. J. Haines R. F. Randle R. J. (1985). “Head-up display (HUD) utility, II: Runway to HUD transitions monitoring eye focus and decision times,” in *Proceedings of the Human Factors Society Annual Meeting*, Vol. 29 Los Angeles, CA: SAGE Publications, 615–619. 10.1177/154193128502900621

[B47] Wickens C. D. (2002). Multiple resources and performance prediction. *Theor. Issues Ergon. sci.* 3 159–177. 10.1080/14639220210123806

[B48] Wiedenmaier S. Oehme O. Schmidt L. Luczak H. (2003). Augmented reality (ar) for assembly processes design and experimental evaluation. *Int. J. Hum. Comput. Int.* 16 497–514. 10.1207/s15327590ijhc1603_7

[B49] Zaeh M. F. Wiesbeck M. Stork S. Schubö A. (2009). A multi-dimensional measure for determining the complexity of manual assembly operations. *Prod. Eng.* 3 489–496. 10.1007/s11740-009-0171-3

